# Systematic review of validated parent-reported questionnaires assessing swallowing dysfunction in otherwise healthy infants and toddlers

**DOI:** 10.1186/s40463-021-00549-3

**Published:** 2021-12-04

**Authors:** Abdulsalam Baqays, Julianna Zenke, Sandra Campbell, Wendy Johannsen, Marghalara Rashid, Hadi Seikaly, Hamdy El-Hakim

**Affiliations:** 1 Division of Otolaryngology-Head and Neck Surgery, Department of Surgery, University of Alberta, 2C3. 57 Walter MacKenzie Center, Edmonton, AB T6H0R3 Canada; 2 John W. Scott Health Sciences Library, University of Alberta, Edmonton, AB Canada; 3 Department of Pediatric Speech Language Pathology, Stollery Children’s Hospital, Edmonton, AB Canada; 4 Department of Pediatrics, University of Alberta, Edmonton, AB Canada; 5 Department of Otolaryngology-Head and Neck Surgery, King Saud University, Riyadh, Saudi Arabia

**Keywords:** Swallowing dysfunction, Dysphagia, Deglutition, Otherwise healthy infants and toddlers, Patient-reported outcomes, Psychometrics, Systematic review

## Abstract

**Objectives:**

There has been increasing interest in the management of oropharyngeal swallowing dysfunction (SwD). Its prevalence, particularly in otherwise healthy infants and toddlers (OHITs), is underappreciated. As the standard diagnostic tests are either invasive or scarce, valid parent-reported outcome (PRO) questionnaires could play a pivotal role in the understanding and managing SwD in this group. This article reviewed the literature on PRO questionnaires pertaining to SwD in OHITs.

**Data source:**

A librarian searched Prospero, Cochrane Library, Embase, Medline, PsycINFO, HaPI, CINAHL, and SCOPUS until February 2021 using the MeSH terms for deglutition and screening methods.

**Review method:**

Questionnaires that examined disease-specific or eating and feeding concerns or difficulties were excluded. Two reviewers independently identified PRO questionnaires for SwD that were used in OHITs and extracted the author names, publication year, questionnaire name, the studied population, and the reported psychometric assessments. A quality assessment was performed based on consensus-based standards for the selection of health measurement instruments (COSMIN) and updated criteria for good measurement properties.

**Results:**

Of the 3488 screened articles, we identified only two questionnaires, the pediatric version of the Eating Assessment Tool (PEDI-EAT-10) and the PRO questionnaire for Swallowing Dysfunction in OHITs. The PEDI-EAT-10 authors assessed the validity and reliability on children with cerebral palsy. However, concerns were identified regarding the developmental process and the internal structure validity. The PRO questionnaire for SwD in OHITs meets criteria but has not yet been validated in the population of interest nor its psychometric properties assessed.

**Conclusion:**

Two instruments were identified. The PED-EAT-10 exhibits methodological flaws, while Edmonton PRO questionnaire for SwD in OHITs awaits construct validation and could fill the current knowledge gap.

**Supplementary Information:**

The online version contains supplementary material available at 10.1186/s40463-021-00549-3.

## Background

The reported prevalence of swallowing dysfunction (SwD) in children is ambiguous. Based on data from national healthcare surveys, it reportedly affects 500,000 children per year in the United States [1]; however, the study this statistic is based on has methodological flaws, including a lower age limit of seven years and non-specific inquiry, that limit extrapolation. Clearer information for decision-makers is required to understand the magnitude of this problem. SwD in otherwise healthy children as a subgroup constitutes between 40 and 90% of the published case series, with a median age at diagnosis of 6.6 months and approximately two years at time of surgery [2–5]. Although these studies have their limitations, they suggest that this cohort represents a major proportion of the children affected.

Both videofluoroscopic swallowing study (VFSS) and functional endoscopic evaluation of swallowing (FEES) are considered gold standard diagnostic tests for SwD [6, 7]. However, these tests are labor-intensive and require expensive specialized equipment in addition to the presence of highly trained personnel. Moreover, VFSS carries risks associated with radiation exposure [8, 9], while FEES is physically intrusive. From another perspective, VFSS and FEES intrinsically cannot gauge symptoms and correlate them to management outcomes, which is a central concept in healthcare. There is potential to narrow this gap with the use of patient-reported outcome (PRO) tools [10], for which there is growing support for their utilization within the pediatric otolaryngology community [11].

Myer et al. published a systematic review (2016) investigating valid PRO questionnaires for pediatric SwD [12]. The review included questionnaires assessing children up to 18 years old and included high-risk groups such as neurologically and anatomically affected children. They identified and evaluated four PRO-based tools [13–16]; however, all of which were disease-specific and had not been clinically validated in SwD among otherwise healthy infants and toddlers (OHITs) who we define as children less than two years of age.

Debate remains regarding the definition of feeding disorders and how it differs from that of dysphagia and swallowing dysfunction. According to the American Speech-Language-Hearing Association 2011, the term “feeding disorder” is a label for disorders where the child has failed to appropriately develop or effectively deploy eating and drinking behaviors, including the placement, manipulation, and movement of the food in the mouth posteriorly [17]. By contrast, dysphagia is considered any interference in the movement of food from the mouth to the stomach [18]. SwD in this context is the oropharyngeal component of dysphagia and is mostly associated with the events of penetration and aspiration. The current review focuses on SwD.

The objective of this study was to perform a comprehensive systematic review of the available literature on PRO questionnaires that assess SwD in OHITs.

## Methods

### Search strategy and terms

The Preferred Reporting Items for Systematic Reviews and Meta-Analyses (PRISMA) protocol was used as a standardized roadmap for conducting this review [19]. In August 2018, a specialized medical librarian performed electronic database searches of Medline, Wiley Cochrane Library, Scopus, EMBASE, PROSPERO, Health and Psychosocial Instruments, and CINAHL. Additionally, ProQuest Dissertations, hand search, grey literature, and review articles were searched for relevant studies. The search strategy included both text words and controlled vocabulary (e.g. MeSH, EMTREE) for the concepts of “deglutition” and “screening methods.” To ensure comprehensive coverage of the literature, the search terms, and references of previous systematic reviews (Hackathon et al., Speyer et al., and Myers et al.) were included [12, 20, 21]. All databases were searched up to August 2018, and retrieved articles were limited to the pediatric population. Key terms, medical headings, and search strategies are outlined in Additional file 1: Table S2. The results were exported to a citation manager (ProQuest RefWorks, 2019), and duplicates were removed prior to screening. An updated search was performed in February 2021.

### Study eligibility, inclusion, and exclusion criteria

All abstracts and full articles addressing SwD assessment scales or questionnaires were eligible for this review. Two independent reviewers assessed and evaluated whether the studies met the eligibility criteria to carry forward to the full article screening phase. A third independent reviewer resolved any disagreement. Assessment tools were included if they were questionnaires specific to SwD that were built based on PRO standards and targeted healthy infants and toddlers, which was defined as children younger than two years of age with no syndromes or related neurological impairments.

The exclusion criteria included all condition-specific questionnaires that addressed neurological conditions, esophageal disease, cardiac-related conditions, and syndromes, or were restricted or targeted to older children. Quality of life questionnaires were also excluded. Reports were also excluded in the screening phase if they did not state the development method that was used.

### Data extraction, quality assessment, and reporting of results

Once agreement on the included studies had been achieved, data extraction was performed independently by two extractors. They followed a pre-specified form that captured author names, publication year, instrument or questionnaire used, characteristics of the study population, psychometric assessment measures, and whether the questionnaire was developed based on PRO guidelines. Psychometric properties were assessed using the consensus‐based standards for the selection of health measurement instruments (COSMIN) [22–24]. The use of COSMIN allowed for a valid assessment of the methodological quality of the included studies. The taxonomy consists of four areas of assessment: reliability, validity, responsiveness, and interpretability.

## Results

**Fig. 1:**
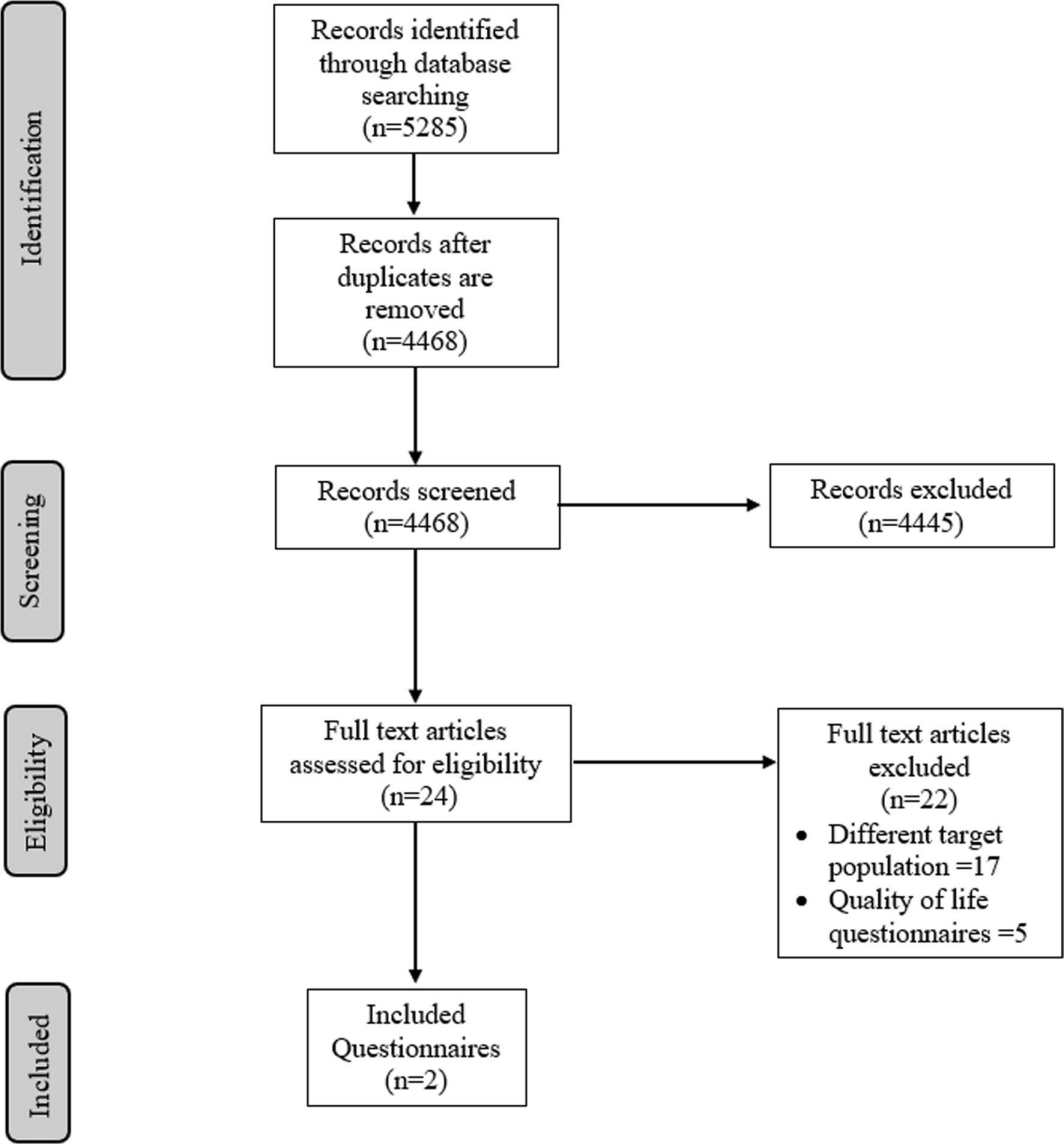
PRISMA diagram detailing the article selection process for further evaluation and inclusion in the systematic review to identify validated PRO questionnaires used for OHITs

The search identified 4468 studies after duplicates were removed. Of those, 24 proceeded to full-text screening (Fig. 1). At this stage, 22 of them met the exclusion conditions [15, 25–45]. Seventeen articles addressed populations with specific conditions or targeted an older age group [25–31, 33–36, 38–40, 43–45]. Five studies were excluded for assessing quality of life (QoL) [15, 32, 37, 41, 42]. Only two studies of the identified 24 studies that underwent full text review described potentially useful tools; however, neither fully met the inclusion criteria of this systematic review. The characteristics and reasons for exclusion of the other 22 tools are described in Additional file 1: Table S3.

The pediatric version of the eating assessment tool (PEDI-EAT-10) [46] was one of two potential tools identified. Its content validity was assessed using a Delphi method. The authors reported the content validity index to be 91%; this index was referred as the sum of CVR means for items. Table 1 presents the characteristics of the PEDI-EAT-10 questionnaire. The report assessed the validity and reliability of the tool in children with cerebral palsy, aged 18 months to 18 years of age.

**Table 1:** Psychometric characteristics of PEDI-EAT-10 questionnaire

Questionnaire	Year	Age	Study population	Study type	Development	Overview
PEDI-EAT-10 ^44^	2017	18 months to 18 years	51 controls and 138 children with spastic cerebral palsy	Cross-sectional	Adapted from the EAT-10 questionnaire and examined in 2 rounds of Delphi technique	10 items of a 4-point scale
	Reliability	Measurement error	Content validity	Hypothesis testing	Criterion validity	Responsiveness
	Excellent test–retest reliability with intraclass correlation coefficient	N/A	Lawshe’s content validity index = 0.91	N/A	The PAS was selected as a related outcome measure and was used to test criterion validity of the PEDI-EAT-10. The excellent correlation between the PEDI-EAT-10 and the scores of PAS suggests that the PEDI-EAT-10 has sufficient criterion validity	N/A

*PEDI-EAT-10* Pediatric Version of the Eating Assessment Tool, *PAS* Penetration–Aspiration Scale

**Fig. 2:**
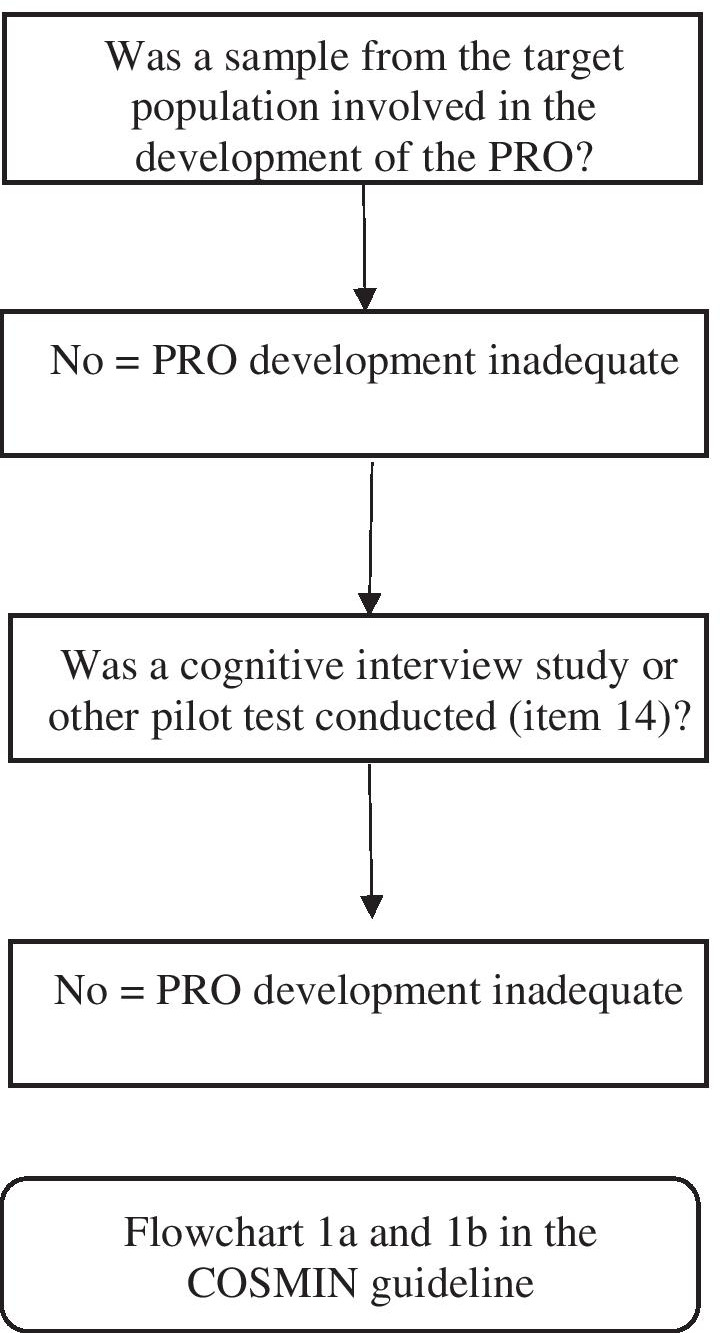
COSMIN flowchart to evaluate the study quality in the development of PEDI-EAT-10

**Fig. 3:**
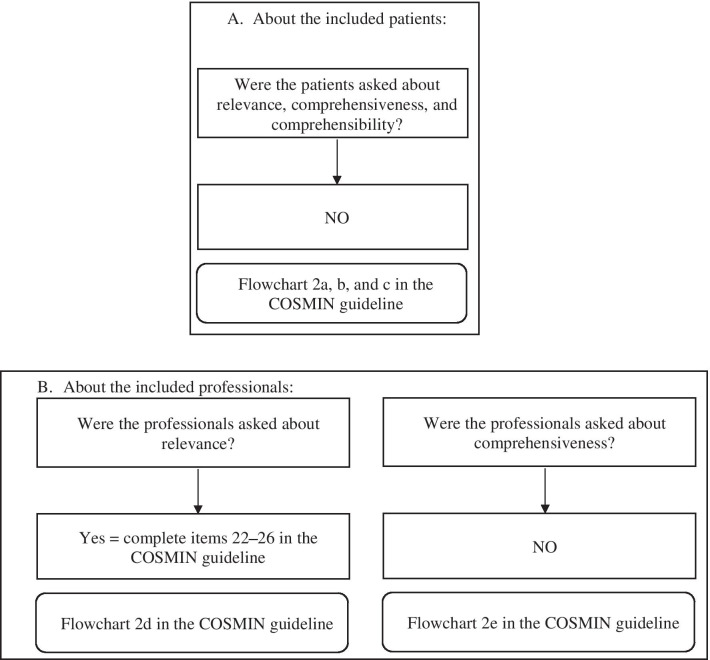
COSMIN flowchart to evaluate the study quality in the content validation of PEDIEAT-10

The PEDI-EAT-10 is an adaptation of the EAT-10 questionnaire [47], a valid tool to assess SwD in adults. Two Delphi rounds were completed with an expert panel of healthcare providers to refine the tool. This questionnaire is a 10-item, caregiver-reported, Likert scale-based instrument that is designed to assess weight gain, ability to eat in public, difficulty swallowing solids or liquids, gaging, pain, desire to eat, choking, coughing, and mealtime stress. The internal consistency (Cronbach’s alpha = 0.87), content validity (content validity index = 0.91), and test–retest reliability were reported for each item as an intraclass correlation coefficient. The study team found a sensitivity of 91.3% and specificity of 98.8% in predicting penetration/aspiration with a score > 4 on the Penetration Aspiration Scale (PAS). Tables outlining the risk of bias checklist for the psychometric properties, content validity internal consistency, reliability, and criterion validity of PEDI-EAT-10 may be found in the Additional file 1. Finally, the COSMIN content validity domain assessment is shown in Figs. 2 and 3.

The Parent-Reported Outcome Questionnaire for Swallowing Dysfunction in Healthy Infants and Toddlers was identified as the only tool that met the inclusion criteria of this systematic review [48]. However, the tool is still in the process of development and while the framework construction and content validity was established, the psychometric properties of the tool have not been established at this time. As such the questionnaire was not assessed using the COSMIN tool.

The study achieved information saturation after conducting 10 parent interviews, generating seven domains with a total of 72 items. Following parent interviews, the authors reported a content validity index of 82.1% after three rounds of a modified Delphi process. The Delphi process reduced the number of domains to three (swallowing, breathing and illness) with a total of 21 items. Content validity was subsequently measured using Lawshe’s content validity ratio for each item with the mean of these values being used to calculate the content validity index (82.1%). However further work is needed regarding psychometric assessment and to establish the construct validity and reliability of the tool. While the items to be used in the tool have been established, the final form of the questionnaire remains to be developed.

## Discussion

Three systematic reviews examined questionnaires that assessed SwD in children [12, 20, 21]. Heckathorn et al. and Speyer et al. aimed to identify non-instrumental assessment tools for feeding and SwD in the pediatric population [20, 21]. This was a broad aim that resulted in including tools that evaluated SwD and feeding (separately or together) and targeted a wide age range (from birth up 18 years). The authors performed their search on two engines only (Medline and EMBASE), which limited their results. Subsequently, Myer et al. took a more focused approach by searching for a validated patient- or proxy parent-reported outcome tool for pediatric SwD (up 18 years) [12]. These authors searched a variety of electronic databases (Scopus, EMBASE, PubMed, Cochrane Library, and CINAHL) [12]. However, as mentioned earlier, none of the four tools they identified were suitable or designed for OHIT’s.

This current systematic review identified the PEDI-EAT-10 [46] as a potential tool. However, the PEDI-EAT-10 [46] has several shortcomings. First, it was adapted from the EAT-10 questionnaire [12, 47] and retained the original conceptual framework of the adult version. Patient engagement is the backbone of the PRO tool construction process; hence, the development of PEDI-EAT-10 deviated from the PRO guidelines. Adapting the questions from a tool previously validated in adults misses the opportunity for parents/patients to contribute. Subsequently, the tool may overlook assessment domains that capture the experience of the caregivers. Second, a test–retest reliability assessment was undertaken to confirm the reliability of PEDI-EAT-10 [46]. But the psychometric properties were assessed on a cohort of children with neurological impairment (cerebral palsy), 90% of whom were grades 2–4 of the Gross Motor Classification System. Furthermore, the minimum age of the group was 18 months. Although the group generated normative data from a trial of the scale on 51 healthy children (age range 18 months to 18 years), the measurement error and responsiveness in otherwise healthy infants and toddlers remain questionable.

A related issue concerns the use of the PAS to ascertain criterion validity. This scale is an objective scale that assesses the severity of SwD based on VFSS [49]. However, this scale is only validated and standardized to assess the severity of SwD in adults. Gosa et al. attempted to establish the reliability of PAS in children by reviewing 25 VFSS studies of a broad age range cohort (mean = 4 years ± 2 months) [49, 50], which was a fairly small study. Most importantly, the quoted intra- and interclass correlations were not from a pediatric population and not from the cohort tested.

Serel et al. applied some changes to PEDI-EAT-10 based on their literature search and expert consensus [46]. Question number five in EAT-10, which inquires about pill swallowing, was replaced by a question about gaging during swallowing. Upon closer inspection and a comparison between the items of both tools, they appeared nearly identical in concept perspective but different in their phrasing. The authors applied minimal linguistic modifications to make the tool usable in children. This is a major flaw of this tool.

The Edmonton PRO questionnaire for Swallowing Dysfunction in Healthy Infants and Toddlers [48] acknowledges the gap in the literature surrounding the assessment of OHITs for SwD. The authors presented a study that adhered to PRO guidelines to identify relevant items related to SwD in this population. However, the questionnaire itself along with an evaluation of its psychometric properties has not yet been published.

The most vital measurement property is content validity. Content validity reflects the clarity, relevancy, and comprehensiveness with respect to the construct of interest (i.e. SwD) and with respect to the target population, which is the pediatric cohort [23]. There is consistent agreement regarding the use of Lawshe’s content validity ratio and index as a quantification method to assess content validity [51, 52]. The authors of both identified studies used the Delphi method. The PEDI-EAT-10 including seven panelists, while the PRO questionnaire for Swallowing Dysfunction in OHITs included nine panelists to extract the ratio and index. However, PEDI-EAT-10 only reported content validity index. Thus, we do not know the agreement ratio of each item, and there was no report of whether all the items passed the CVR threshold or not. Conversely, the PRO questionnaire for Swallowing Dysfunction in Healthy Infants and Toddlers reports the agreement ratio for each item along with whether the items passed the CVR ratio.

Moreover, the PRO questionnaire for Swallowing Dysfunction in OHITs expands beyond the PEDI-EAT-10 which focuses solely on the domain of swallowing, to further include items related to breathing and illness. While both tools overlap in the domain of swallowing, there is little overlap between individual items, thus establishing both tools as unique in content and application.

Our review was constructed to identify a standardized assessment tool for SwD that was specific to OHITs. Although the literature on the epidemiology of SwD in healthy infants and toddlers is scant, the reported parameters of some cross-sectional case series have drawn attention to this group. In the studies by Sheikh [2], Syvstun [4], and Lefton-Greif [3] and their coauthors (greater than 3 to 4 years of management at tertiary care facilities albeit diverse settings), the reported mean ages were 2 ± 1.6 months, 6.6 months (range 3.1–17.1 months), and 1.14 years (range 0.9–5.75), respectively. Two of these reports were based on limited sample sizes of healthy children diagnosed with SwD during the course of investigating unidentified respiratory problems [2, 3]. The series reported by Svysten et al. analyzed over 170 consecutive children managed at a multidisciplinary swallowing practice, and nearly 75% of them did not have comorbidities known to be associated with SwD [4].

Further, upon examining a surgical case series, the mean ages at laryngeal cleft repair or injection laryngoplasty were 24.7 months (range 4–63) [53], 25.3 months (range 2–120) [5], and 1.6 years [54]. Bearing in mind that conservative measures had been adopted for several months as an initial step, one can conclude that the mean age at diagnosis was close to that in the previously described series. In the above respective studies, thirteen of 20, twenty of 54, and nine of 22 children were otherwise neurologically healthy.

The cited sources are mostly retrospective studies and some report select groups; therefore, these studies harbor methodological flaws. Yet, they all indicate that otherwise children, particularly those within the first 2–3 years of life, are a sizable proportion of children with SwD who require active management. As yet, we do not have a validated PRO tool to supplement detection and diagnosis in this cohort.

## Conclusion

This systematic review identified two potential tools to assess SwD in OHITs. However, one was not constructed according to PRO methodology nor was it applied to the population of interest and the other has not yet been validated or its psychometric properties reported on. The findings of the review will guide future studies in overcoming the methodological flaws of current tools and further contribute to the development of a PRO questionnaire validated in OHITs.

## Supplementary Information

**Additional file 1: Table S2.** Characteristics of and reasons for the excluded studies.

## Data Availability

Data sharing is not applicable to this article as no datasets were generated or analysed during the current study.

## Abbreviations

SwD: Swallowing dysfunction
OHIT: Otherwise healthy infants and toddlers
PRO: Patient/parent-reported outcomes
VFSS: Videofluoroscopic swallowing study
FEES: Function endoscopic evaluation of swallowing
