# Supplementary material for: Systematic review of validated parent-reported questionnaires assessing swallowing dysfunction in otherwise healthy infants and toddlers
Source: J Otolaryngol Head Neck Surg. 2021 Dec 4;50:68. doi: 10.1186/s40463-021-00549-3 (PMC8642864; doi:10.1186/s40463-021-00549-3)
Supplement: Supplementary file 1 — Additional file 1: Table S2. Characteristics of and reasons for the excluded studies. [file 40463_2021_549_MOESM1_ESM.pdf]

Table S2. Search methodology and strategies.

Search engine	Search Strategy
1-Ovid MEDLINE(R) and Epub (1946 to August 17, 2018)	1. exp child/ or exp "congenital, hereditary, and neonatal diseases and abnormalities"/ or exp infant/ or adolescent/ or exp pediatrics/ or child, abandoned/ or exp child, exceptional/ or child, orphaned/ or child, unwanted/ or minors/ or (pediatric* or paediatric* or child* or newborn* or congenital* or infan* or baby or babies or neonat* or pre-term or preterm* or premature birth* or NICU or preschool* or pre-school* or kindergarten* or kindergarden* or elementary school* or nursery school* or (day care* not adult*) or schoolchild* or toddler* or boy or boys or girl* or middle school* or pubescen* or juvenile* or teen* or youth* or high school* or adolesc* or pre-pubesc* or prepubesc*).mp. or (child* or adolesc* or pediat* or paediat*).jn. (4702627)<br>2. ("functional outcome swallowing scale" or "functional oral intake scale" or FOIS or "eating assessment tool" or "swallowing quality of life questionnaire" or ((dysphagia or deglutition) and "handicap index") or "videofluoroscopic dysphagia scale" or "clinical dysphagia scale" or "American Speech Language Hearing Association's National Outcome Measurement System swallowing scale" or "Anderson Dysphagia Inventory" or "European dysphagia group questionnaire" or "Assessment Evaluation and Programming System for Infants and Children" or BAMBI or "Brief Autism Mealtime Behavior Inventory" or BAMF OMD or BAMFOMD or "Oral Motor Deglutition scale" or BASOFF or "Behavioral assessment scale of oral functions in feeding" or "Bedside Evaluation of Dysphagia" or "Colorado Childhood Temperament Inventory" or "Children's Eating Behavior Inventory" or "Children's Eating Behavior Questionnaire" or "Child Feeding Questionnaire" or "Child Mealtime Feeding Behavior Questionnaire" or "Developmental Assessment for Individuals with Severe Disabilities" or "Developmental Assessment of Young Children" or DYMUS or "Dysphagia in Multiple Sclerosis" or "Dysphagia Symptom Questionnaire" or "PedsQL GI Module" or "Pediatric Quality of Life Inventory Gastrointestinal Symptoms Moduleor Dysphagia Evaluation Protocol" or "Dysphagia Disorder Survey or Dysphagia Disorders Survey" or "Dyadic Interaction

Nomenclature for Eating" or "Drooling Severity and Frequency Scale" or "Early Feeding Skills Assessment" or "Family Environment Scale" or "Frenchay Dysarthria Assessment" or "Feeding and Swallowing Questionnaire" or "Feeding Strategies Questionnaire" or "Gisel Video Assessment " or "Infant Feeding Style Questionnaire" or "Infant Toddler and Family Instrument" or "Multidisciplinary Feeding Profile" or "Neonatal Oral Motor Assessment Scale" or "Oral Assessment Guide for children and young people" or "Oropharyngeal Dysphagia" or "Oral Motor Assessment Scale" or PASSFP or "Pediatric Assessment Scale for Severe Feeding Problems" or PIBBS or "Preterm Infant Breastfeeding Behavior Scale" or "Parent Mealtime Action Scale" or "Swallowing Ability and Function Evaluation " or "Systematic Assessment of the Infant at Breast" or "Schedule for Oral Motor Assessment" or "Child Screening Tool of Feeding Problems" or "SWAL QoL" or "Swallowing Quality of Life Questionnaire" or ((AEPS or CCTI or STEP or SAIB or PMAS or OMAS or IFTI or OAG or MFP or "FDA 2" or IFSQ or SOMA or SAFE or FSQ or FES or EFS or DSQ or GVA or DSFS or "DAYC 2" or o DINE DEP or NOMAS or "DASH 3" or CFQ or CEBI or CEBQ or CCTI or CMFBQ) adj2 (scale or questionnaire or survey or inventory or assessment or protocol or screen* or evaluation))).mp. [mp=title, abstract, original title, name of substance word, subject heading word, floating sub-heading word, keyword heading word, protocol supplementary concept word, rare disease supplementary concept word, unique identifier, synonyms] (8974)

3. (index or inventory or protocol or profile or test* or tool* or screening or screened or questionnaire* or checklist* or survey* or instrument* or evaluation method*).ti. [mp=title, abstract, original title, name of substance word, subject heading word, floating sub-heading word, keyword heading word, protocol supplementary concept word, rare disease supplementary concept word, unique identifier, synonyms] (931445)
4. Validation Studies/ or validation stud*.mp. or "Surveys and Questionnaires"/ or "Checklist"/ or clinical assessment tool.mp. or psychometrics/ or psychometric*.mp. [mp=title, abstract, original title, name of substance word, subject heading word, floating sub-heading word, keyword heading

	word, protocol supplementary concept word, rare disease supplementary concept word, unique identifier, synonyms] (547819)<br>5. 2 or 3 or 4 (1384080)<br>6. exp DEGLUTITION DISORDERS/ or exp Deglutition/ or deglutition.mp. or swallow*.mp. or dysphagia.mp. (81719)<br>7. 1 and 5 and 6 (1315)<br>8. remove duplicates from 7 (1312)
2-Embase (1974 to August 20, 2018)	1. *"functional outcome swallowing scale"/ or ("functional outcome swallowing scale" or "functional oral intake scale" or FOIS or "eating assessment tool" or "swallowing quality of life questionnaire" or ((dysphagia or deglutition) and "handicap index") or "videofluoroscopic dysphagia scale" or "clinical dysphagia scale" or "American Speech Language Hearing Association's National Outcome Measurement System swallowing scale" or "Anderson Dysphagia Inventory" or "European dysphagia group questionnaire" or "Assessment Evaluation and Programming System for Infants and Children" or BAMBI or "Brief Autism Mealtime Behavior Inventory" or BAMF OMD or BAMFOMD or "Oral Motor Deglutition scale" or BASOFF or "Behavioral assessment scale of oral functions in feeding" or "Bedside Evaluation of Dysphagia" or "Colorado Childhood Temperament Inventory" or "Children's Eating Behavior Inventory" or "Children's Eating Behavior Questionnaire" or "Child Feeding Questionnaire" or "Child Mealtime Feeding Behavior Questionnaire" or "Developmental Assessment for Individuals with Severe Disabilities" or "Developmental Assessment of Young Children" or DYMUS or "Dysphagia in Multiple Sclerosis" or "Dysphagia Symptom Questionnaire" or "PedsQL GI Module" or "Pediatric Quality of Life Inventory Gastrointestinal Symptoms Moduleor Dysphagia Evaluation Protocol" or "Dysphagia Disorder Survey or Dysphagia Disorders Survey" or "Dyadic Interaction Nomenclature for Eating" or "Drooling Severity and Frequency Scale" or "Early Feeding Skills Assessment" or "Family Environment Scale" or "Frenchay Dysarthria Assessment" or "Feeding and Swallowing Questionnaire" or "Feeding Strategies Questionnaire" or "Gisel Video Assessment " or "Infant Feeding Style Questionnaire" or "Infant Toddler and Family Instrument" or "Multidisciplinary Feeding Profile" or "Neonatal Oral

Motor Assessment Scale" or "Oral Assessment Guide for children and young people" or "Oropharyngeal Dysphagia" or "Oral Motor Assessment Scale" or PASSFP or "Pediatric Assessment Scale for Severe Feeding Problems" or PIBBS or "Preterm Infant Breastfeeding Behavior Scale" or "Parent Mealtime Action Scale" or "Swallowing Ability and Function Evaluation " or "Systematic Assessment of the Infant at Breast" or "Schedule for Oral Motor Assessment" or "Child Screening Tool of Feeding Problems" or "SWAL QoL" or "Swallowing Quality of Life Questionnaire" or ((AEPS or CCTI or STEP or SAIB or PMAS or OMAS or IFTI or OAG or MFP or "FDA 2" or IFSQ or SOMA or SAFE or FSQ or FES or EFS or DSQ or GVA or DSFS or "DAYC 2" or o DINE DEP or NOMAS or "DASH 3" or CFQ or CEBI or CEBQ or CCTI or CMFBQ) adj2 (scale or questionnaire or survey or inventory or assessment or protocol or screen* or evaluation))).mp. [mp=title, abstract, heading word, drug trade name, original title, device manufacturer, drug manufacturer, device trade name, keyword, floating subheading word, candidate term word] (12637)

2. (index or inventory or protocol or profile or test* or tool* or screening or screened or questionnaire* or checklist* or survey* or instrument* or evaluation method*).ti. [mp=title, abstract, heading word, drug trade name, original title, device manufacturer, drug manufacturer, device trade name, keyword, floating subheading word, candidate term word] (1072570)
3. validation study/ or validation stud*.mp. or *"clinical evaluation"/ or diagnostic test accuracy study/ or checklist/ or clinical assessment tool/ or screening/ or clinical assessment tool.mp. or "assessment of humans"/ or rating scale/ or scoring system/ or questionnaire/ or *functional assessment/ or psychometry/ or psychomet*.mp. [mp=title, abstract, heading word, drug trade name, original title, device manufacturer, drug manufacturer, device trade name, keyword, floating subheading word, candidate term word] (1238244)
4. 1 or 2 or 3   (2118996)
5. exp DEGLUTITION DISORDERS/ or exp Deglutition/ or deglutition.mp. or swallow*.mp. or dysphagia.mp. (98129)
6. exp swallowing/ or exp dysphagia/ (74190)
7. 5 or 6 (98129)

	8.juvenile/ or exp adolescent/ or exp child/ or exp postnatal development/ or (pediatric* or paediatric* or child* or newborn* or congenital* or infan* or baby or babies or neonat* or pre-term or premature birth or NICU or preschool* or pre-school* or kindergarten* or elementary school* or nursery school* or schoolchild* or toddler* or boy or boys or girl* or middle school* or pubescen* or juvenile* or teen* or youth* or high school* or adolesc* or pre-pubesc*).mp. or (child* or adolesc* or pediat* or paediat*).jn. (4195306)<br>9.4 and 7 and 8 (1749)<br>10. remove duplicates from 9 (1733)
3- PsycINFO (1806 to August Week 2, 2018)	1.("functional outcome swallowing scale" or "functional oral intake scale" or FOIS or "eating assessment tool" or "swallowing quality of life questionnaire" or ((dysphagia or deglutition) and "handicap index") or "videofluoroscopic dysphagia scale" or "clinical dysphagia scale" or "American Speech Language Hearing Association's National Outcome Measurement System swallowing scale" or "Anderson Dysphagia Inventory" or "European dysphagia group questionnaire" or "Assessment Evaluation and Programming System for Infants and Children" or BAMBI or "Brief Autism Mealtime Behavior Inventory" or BAMF OMD or BAMFOMD or "Oral Motor Deglutition scale" or BASOFF or "Behavioral assessment scale of oral functions in feeding" or "Bedside Evaluation of Dysphagia" or "Colorado Childhood Temperament Inventory" or "Children's Eating Behavior Inventory" or "Children's Eating Behavior Questionnaire" or "Child Feeding Questionnaire" or "Child Mealtime Feeding Behavior Questionnaire" or "Developmental Assessment for Individuals with Severe Disabilities" or "Developmental Assessment of Young Children" or DYMUS or "Dysphagia in Multiple Sclerosis" or "Dysphagia Symptom Questionnaire" or "PedsQL GI Module" or "Pediatric Quality of Life Inventory Gastrointestinal Symptoms Moduleor Dysphagia Evaluation Protocol" or "Dysphagia Disorder Survey or Dysphagia Disorders Survey" or "Dyadic Interaction Nomenclature for Eating" or "Drooling Severity and Frequency Scale" or "Early Feeding Skills Assessment" or "Family Environment Scale" or "Frenchay Dysarthria Assessment" or "Feeding and Swallowing Questionnaire" or "Feeding Strategies Questionnaire" or "Gisel Video

Assessment " or "Infant Feeding Style Questionnaire" or "Infant Toddler and Family Instrument" or "Multidisciplinary Feeding Profile" or "Neonatal Oral Motor Assessment Scale" or "Oral Assessment Guide for children and young people" or "Oropharyngeal Dysphagia" or "Oral Motor Assessment Scale" or PASSFP or "Pediatric Assessment Scale for Severe Feeding Problems" or PIBBS or "Preterm Infant Breastfeeding Behavior Scale" or "Parent Mealtime Action Scale" or "Swallowing Ability and Function Evaluation " or "Systematic Assessment of the Infant at Breast" or "Schedule for Oral Motor Assessment" or "Child Screening Tool of Feeding Problems" or "SWAL QoL" or "Swallowing Quality of Life Questionnaire" or ((AEPS or CCTI or STEP or SAIB or PMAS or OMAS or IFTI or OAG or MFP or "FDA 2" or IFSQ or SOMA or SAFE or FSQ or FES or EFS or DSQ or GVA or DSFS or "DAYC 2" or o DINE DEP or NOMAS or "DASH 3" or CFQ or CEBI or CEBQ or CCTI or CMFBQ) adj2 (scale or questionnaire or survey or inventory or assessment or protocol or screen* or evaluation))).mp. [mp=title, abstract, heading word, table of contents, key concepts, original title, tests & measures] (4926)

2. exp Test Validity/ or exp Psychometrics/ (95879)
3. exp SURVEYS/ (8806)
4. exp "Checklist (Testing)"/ or exp Test Reliability/ or exp SYMPTOM CHECKLISTS/ or exp Rating Scales/ (67679)
5. (instrument or instruments or indicies or index* or inventory or inventories or scale or scales or screen or screened or screening or surve* or checklist* or questionnaire or protocol* or assessment* or evaluat* or tool or tools).mp. (1762574)
6. clinical assessment tool*.mp. (159)
7. 1 or 2 or 3 or 4 or 5 or 6 (1772399)
8. exp swallowing/ or exp dysphagia/ or (swallow* or dysphagia* or deglutition*).mp. (4089)
9. adolescent development/ or childhood development/ (101763)
10. pediatrics/ (22725)
11. exp Congenital Disorders/ (7416)
12. child characteristics/ (2102)
13. chronically ill children/ (315)

	14. child abuse/ or exp child welfare/ (34347)<br>15. child neglect/ (3810)<br>16. child psychiatry/ or child psychopathology/ (9051)<br>17. exp child care/ (9160)<br>18. (pediatric* or paediatric* or child* or newborn* or congenital* or infan* or baby or babies or neonat* or pre-term or preterm* or premature birth* or NICU or preschool* or pre-school* or kindergarten* or kindergarden* or elementary school* or nursery school* or (day care* not adult*) or schoolchild* or toddler* or boy or boys or girl* or middle school* or pubescen* or juvenile* or teen* or youth* or high school* or adolesc* or pre-pubesc* or prepubesc*).mp. or (child* or adolesc* or pediat* or paediat*).jn. (1071265)<br>19. 9 or 10 or 11 or 12 or 13 or 14 or 15 or 16 or 17 or 18 (1072711)<br>20. 7 and 8 and 19 (363)<br>21. remove duplicates from 20 (363)
4- Health and Psychosocial Instruments (1985 to July 2018)	1. deglutition.mp. [mp=title, acronym, descriptors, measure descriptors, sample descriptors, abstract, source] (20)<br>2. dysphagia.mp. [mp=title, acronym, descriptors, measure descriptors, sample descriptors, abstract, source] (73)<br>3. oropharyn*.mp. [mp=title, acronym, descriptors, measure descriptors, sample descriptors, abstract, source] (23)<br>4. swallow*.mp. [mp=title, acronym, descriptors, measure descriptors, sample descriptors, abstract, source] (74)<br>5. 1 or 2 or 3 or 4 (133)
5- Prospero (August 21, 2018)	1. Deglutination or swallow* or dysphagia (177)<br>2. MeSH DESCRIPTOR Deglutination Disorders EXPLODE ALL TREES (98)<br>3. MeSH DESCRIPTOR Deglutination EXPLODE ALL TREES (34)<br>4. #1 OR #2 OR #3 (228)<br>5. Pediatric* or paediatric* or child or newborn* or congenital* or infan* or baby or babies or neonat* or pre-term or preterm* or premature birth* or NICU or preschool* or pre-school* or kindergarten* or kindergarden* or elementary school* or nursery school* or (day care* not adult*) or schoolchild* or toddler* or boy or boys or girl* or middle school or

	pubescent* or juvenile* or teen* or youth* or high school* or adolesc* or pre-pubesc* (14727)<br>6. Score or scoring or instrument or instruments or indices or index* or inventory or inventories or scale of scales or screen or screened or screening or surve* or checklist* or questionnaire or protocol* or assessment* or evaluat* or tool or tools (39002)<br>7. #6 AND #5 AND #4 (101)
6-CINAHL Plus Full Text (August 21, 2018)	1. ("functional outcome swallowing scale" or "functional oral intake scale" or FOIS or "eating assessment tool" or "swallowing quality of life questionnaire" or ((dysphagia or deglutination) and "handicap index") or "videofluoroscopic dysphagia scale" or "clinical dysphagia scale" or "American Speech Language Hearing Assosciation's National Outcome Measurement System swallowing scale" or "Anderson Dysphagia Inventory" or "European dysphagia group questionnaire" or "Assessment Evaluation and Programmin (4,094)<br>2. "clinical assessment tool" (118,146)<br>3. (TI index or inventory or inventories or protocol* or profile* or test* or tool* or screening or screened or questionnaire* or checklist* or survey* or instrument* or "evaluation method" or score or scoring) (1,575,788)<br>4. (MH "Validation Studies") OR "validation studies" or (MH "Psychometrics") OR (MH "Measurement Issues and Assessments") or psychometric* (112,969)<br>5. (MH "Clinical Assessment Tools") OR (MH "Behavior Rating Scales") OR (MH "Checklists") OR (MH "Questionnaires+") OR (MH "Scales") (547,238)<br>6. (MH "Instrument Validation") (30,006)<br>7. S1 OR S2 OR S3 OR S4 OR S5 OR S6 (1,638,726)<br>8. (pediatric* or paediatric* or child* or newborn* or congenital* or infan* or baby or babies or neonat* or "pre-term" or preterm or "premature birth" or NICU or preschool* or "pre-school*" or kindergarten* or "elementary school*" or "nursery school*" or schoolchild* or toddler* or boy or boys or girl* or "middle school*" or pubescen* or juvenile* or teen* or youth* or "high school*" or adolesc* or prepubesc* or "pre-pubesc*" or "(MH

	“Child+”) OR (MH “Adolescence+”) OR (MH “Minors (Legal)”) or “(M (882,458)<br>9. TI deglutination or swallow* or dysphagia (5351)<br>10. (MH “Deglutination”) OR (MH “Deglutination Disorders”) OR (MH “Swallowing Therapy”)<br>11. S9 OR S10 (9787)<br>12. S7 AND S8 AND S11 (475)<br>13. S7 AND S8 AND S11 (104)<br>14. S7 AND S8 AND S11 (393)<br>15. S13 AND S14 (91)<br>16. S13 NOT S15 (13)<br>17. S12 NOT S16 (462)
7- Cochrane Library (August 23, 2018)	1. (deglutition or swallow* or dysphagia):ti,ab,kw AND (instrument or instruments or indicies or index* or inventory or inventories or scale or scales or screen or screened or screening or surve* or checklist* or questionnaire or protocol* or assessment* or evaluat* or tool or tools):ti AND (pediatric* or paediatric* or child* or newborn* or congenital* or infan* or baby or babies or neonat* or "pre-term" or preterm* or "premature birth*" or NICU or preschool* or "pre-school*" or kindergarten* or kindergarden* or "elementary school*" or "nursery school*" or ("day care*" not adult*) or schoolchild* or toddler* or boy or boys or girl* or "middle school* or pubescen*" or juvenile* or teen* or youth* or "high school*" or adolesc* or "pre-pubesc*" or prepubesc*):ti,ab,kw
8- SCOPUS Searched August 22, 2018	1. (TITLE (pediatric* OR paediatric* OR child* OR newborn* OR congenital* OR infan* OR baby OR babies OR neonat* OR "pre-term" OR preterm* OR "premature birth*" OR nicu OR preschool* OR "preschool*" OR kindergarten* OR kindergarden* OR "elementary school*") OR TITLE ("nursery school*" OR ("daycare*" not AND adult*) OR schoolchild* OR toddler* OR boy OR boys OR girl* OR "middle school*" OR pubescen* "or juvenile* or teen* or youth* or " high AND school* "or adolesc* or " pre-pubesc*" or prepubesc*) OR TITLE(" nursery AND school* " or (" day AND care* " not adult*) or schoolchild* or toddler* or boy or boys or girl* or " middle AND school* " or pubescen*" OR juvenile* OR teen* OR youth* OR "high

	school*" OR adolesc* OR "prepubesc*" OR prepubesc*) AND TITLE (deglutition OR swallow* OR dysphagia) AND TITLE (instrument OR instruments OR indicies OR index* OR inventory OR inventories OR scale OR scales OR screen OR screened OR screening OR surve* OR checklist* OR questionnaire OR protocol* OR assessment* OR evaluat* OR tool OR tools))

Table S3: Characteristics of and reasons for the excluded studies.

Author	Year	Population Age	Study Population	Tool	Reason for Exclusion
Bakke et al.[25]	2007	3–86 years	51 controls and 138 children with spastic cerebral palsy	Nordic Orofacial Test-Screening (NOT-S)	• Older study population.<br>• Neurologically impaired population.<br>• Non-PRO tool.<br>• Clinical assessment tool.
De Felicio et al.[26]	2008	6–12 years	80 children without communication or orofacial myofunctional disorder	• Traditional Orofacial Myofunctional Evolution (TOME)<br>• Orofacial Myofunctional Evaluation with Score (OMES)	• Older study population.<br>• Non-PRO tool.<br>• Clinical assessment tool.
Kamide et al.[27]	2015	2 months to 14 years	54 pediatric patients with dysphagia	Ability for Basic Feeding and Swallowing Scale (ABFS-C)	• Older study population.<br>• Mixed population with comorbidities that affect swallowing.<br>• Clinical assessment tool.
Kendall et al.[28]	2016	16–100 years	139 consecutive patients with dysphagia	Correlation between Eating Assessment Tool (EAT-10) Questionnaire and VFSS	• Older study population.<br>• Non-PRO tool.
Ko et al.[29]	2011	6–48 months	33 children with dysphagia	Schedule for Oral-Motor Assessment	• Non-PRO tool.<br>• Clinical assessment tool.<br>• Mixed population with comorbidities that affect swallowing.
Kwiatek et al.[30]	2011	18–83 years	211 patients with globus sensation	Esophageal Symptoms Questionnaire (ESQ)	• Different aim of the study.<br>• Older study population.<br>• Non-PRO tool.
Lee et al.[31]	2017	<32–37 weeks	52 infants with suspected dysphagia	Dysphagia Screening Test for	• Clinical assessment tool.<br>• Non-PRO tool.

				Preterm Infants (DST-PI)	
Lefton-Greif et al.[32]	2014	Median age 14 months	164 primary caregivers of children presented for feeding/swallowing evaluation	Feeding/Swallowing Impact Survey (FS-IS)	• QoL assessment tool.
Viviers et al.[33]	2017	32 weeks to 4 months	20 neonates	Neonatal Feeding Assessment Scale (NFAS)	• Older study population.<br>• Mixed population with comorbidities that affect swallowing.<br>• Different aim of the study.<br>• Non-PRO tool.
Viviers et al.[34]	2016	No participants	5 expert speech and language pathologists with 5–20 years of experience	Neonatal Feeding Assessment Scale (NFAS)	• Non-PRO tool.<br>• Different aim of the study.
Sarah Monks.[35]	2017	1–3 years	30 families of children having dysphagia with aspiration	Correlation of VFSS score to the clinical symptoms	• Non-PRO tool.<br>• Clinical assessment tool.<br>• Different aim of the study.<br>• Older study population.<br>• Unclear characteristics of the cohort
Moon et al.[36]	2017	27.3 weeks mean gestational age	130 preterm infants who underwent VFSS	Feeding and Swallowing Scale for Premature Infants (FSSPI)	• Non-PRO tool.<br>• Clinical assessment tool.
Erin Redle.[37]	2007	12 months to 4 years	20 primary caregivers in the first phase<br>90 primary caregivers of children with feeding and swallowing disorders	Pediatric Feeding and Swallowing Disorders Family Impact Scale (PFSDFIS)	• QoL assessment tool.<br>• Older study population.<br>• Mixed population with comorbidities that affect swallowing.
Skuse et al.[38]	1995	8–24 months	127 nonorganic failure to thrive and cerebral palsy cases	Schedule for Oral–Motor Assessment (SOMA)	• Different aim of the study.<br>• Non-PRO tool.<br>• Neurologically impaired children.
Sonies et al.[39]	2009	6 months to 20 years	18 participants purposefully selected	Brief Assessment of Motor Function	• Non-PRO tool.<br>• Clinical assessment tool.

				Oral Motor Articulation and Deglutition Scales	
Thoyre et al.[40]	2005	No participants	No participants	Early Feeding Skills (EFS)	• Non-PRO tool.<br>• Clinical assessment tool.
Varni et al.[41]	2015	2–18 years	689 patient families and 552 healthy families	PedsQL Gastrointestinal Symptoms and Worry Scales	• QoL assessment tool.<br>• Older study population.<br>• Different aim of the study.
Varni et al.[42]	2014	2–18 years	689 families	PedsQL Gastrointestinal Symptoms	• QoL assessment tool.<br>• Older study population.<br>• Different aim of the study.
Varni et al.[15]	2012	2–18 years	98 participants with gastrointestinal disorders	PedsQL Gastrointestinal Symptoms	• QoL assessment tool.<br>• Older study population.<br>• Different aim of the study.
Ramsay et al.[43]	2011	Normative sample mean age is 31 months; Clinical nonmedical sample mean age is 24 months; Clinical medical mean age is 26 months	198 normative sample, 91 with clinical nonmedical feeding problem, and 83 with clinical feeding problems	Montreal Children's Hospital Feeding Scale	• Non-PRO tool.<br>• Older study population.<br>• Different aim of the study.
